# Dysfunctional myeloid states in sepsis across the marrow-to-blood continuum: developmental contribution and peripheral reprogramming

**DOI:** 10.3389/fimmu.2026.1967804

**Published:** 2026-10-08

**Authors:** Jiaan Chen, Fan Zhang, Guangjun Jin, Jiancheng Zhang

**Affiliations:** Department of Emergency, The Second Affiliated Hospital of Zhejiang Chinese Medical University, Hangzhou, China

**Keywords:** hematopoietic stem and progenitor cells, immune reprogramming, immunoparalysis, monocytes, myeloid-derived suppressor cells, myelopoiesis, neutrophils, sepsis

## Abstract

Sepsis is accompanied by extensive expansion and remodeling of the myeloid compartment, yet a dysfunctional circulating phenotype does not by itself reveal where that dysfunction arose. Immature markers, low-density fractions, HLA-DR-low phenotypes, transcriptional clusters, and direct T-cell suppression describe different biological levels and carry different mechanistic implications. This review examines sepsis-associated myeloid dysfunction as a source-attribution problem across a marrow-to-blood continuum. Clinical-sepsis studies support altered hematopoietic output and lineage-specific maturation, including emergency-granulopoietic programs in circulating hematopoietic stem and progenitor cells, whereas controlled human systemic endotoxemia provides separate cross-compartment evidence of impaired monocyte maturation. *In vitro*, plasma from patients with sepsis can program healthy human progenitors toward suppressive myeloid states. Conversely, endotoxin tolerance, epigenetic and metabolic remodeling, activation, and degranulation can generate additional dysfunction after marrow egress. Several human observations argue against a unitary maturation-failure model. Mature CD10-positive neutrophils can suppress T-cell proliferation even when immature CD10-negative cells do not; low-density neutrophils encompass mixed maturity and activation states, and immature neutrophils are kinetically distinct from LOX-1-positive polymorphonuclear myeloid-derived suppressor cell (PMN-MDSC)-like populations. Monocyte HLA-DR provides a further example because it functions as a dynamic immune-state readout rather than a measure of developmental maturity. Accordingly, we distinguish five evidentiary layers - hematopoietic origin, developmental state, molecular state, functional phenotype, and patient-level immune phenotype - and use them to reassess neutrophil, monocyte, and MDSC-like states. Current human evidence supports contributions from developmental remodeling and peripheral reprogramming, but their relative importance within the same lineage and patient remains unresolved. Resolving that question will require paired compartment sampling, serial multi-omics, direct functional validation, and clinically meaningful longitudinal outcomes rather than greater reliance on computational trajectories or marker-defined identities alone.

## 1 Introduction

Sepsis is defined clinically by life-threatening organ dysfunction caused by a dysregulated host response to infection, but this definition does not specify which immune program predominates in an individual patient (1). Human profiling studies now show that the heterogeneity is not simply temporal: hyperinflammatory, low-response, myeloid-dominant and mixed states can coexist across compartments and change over days (2–4). In the myeloid compartment, this creates a specific interpretive problem. The label ‘myeloid dysfunction’ may refer to altered cell production, incomplete or diverted maturation, acquisition of a transcriptional state, defective effector function, or a patient-level endotype. These phenomena may occur together, but they describe different biological events (**Box 1**).

**BOX 1** | MDSC: a phenotypically defined myeloid population with direct suppressive-function evidence. MDSC-like: a population with phenotypic or transcriptomic resemblance to MDSC but without direct suppression shown in the same defined population. PMN-MDSC-like: a granulocytic/PMN phenotype compatible with PMN-MDSC when functional validation is incomplete. M-MDSC-like: a monocytic phenotype compatible with M-MDSC when functional validation is incomplete. LDN: a low-density neutrophil defined by density separation; LDN is not synonymous with PMN-MDSC or developmental immaturity.

The unresolved issue is how these increasingly detailed cell states should be interpreted mechanistically. Single-cell studies identify MS1 monocytes, HLA-DR-low/S100A-high monocytes, IL1R2-positive immature neutrophils and other transcriptional states, while conventional assays identify low-density neutrophils (LDNs), reduced monocyte HLA-DR and phenotypically defined myeloid-derived suppressor cells (MDSCs) (5–8). These measurements are informative, but assigning a circulating state to altered marrow maturation requires evidence beyond its presence in blood. An immature-marker-positive neutrophil may have left the marrow early; a mature neutrophil may acquire suppressive function after exposure to the septic milieu; and a monocyte may reduce HLA-DR through transcriptional regulation without being developmentally immature. The blood phenotype alone does not identify the source of dysfunction.

Recent reviews have already covered emergency granulopoiesis, single-cell-defined low-response states, MDSC biology, PBMC trajectories, broad immune reprogramming and phenotype-guided immunotherapy (4, 9–14). The unresolved issue is not another catalogue of these states, but source attribution: what evidence is required before a dysfunctional circulating myeloid state can be assigned to altered production or maturation rather than to post-egress state acquisition? This review therefore focuses on the evidentiary transition from cell description to mechanistic attribution.

We use a ‘marrow-to-blood continuum’ as an organizing device, not as evidence that a continuous human lineage trajectory has been demonstrated. Evidence is considered at five levels: hematopoietic origin, developmental state, molecular state, functional phenotype and patient-level immune phenotype (**Figure 1**). Keeping these levels separate helps reconcile apparently conflicting studies and makes clear where an interpretation rests on direct human data and where it still depends on biological inference.

**Figure 1:**
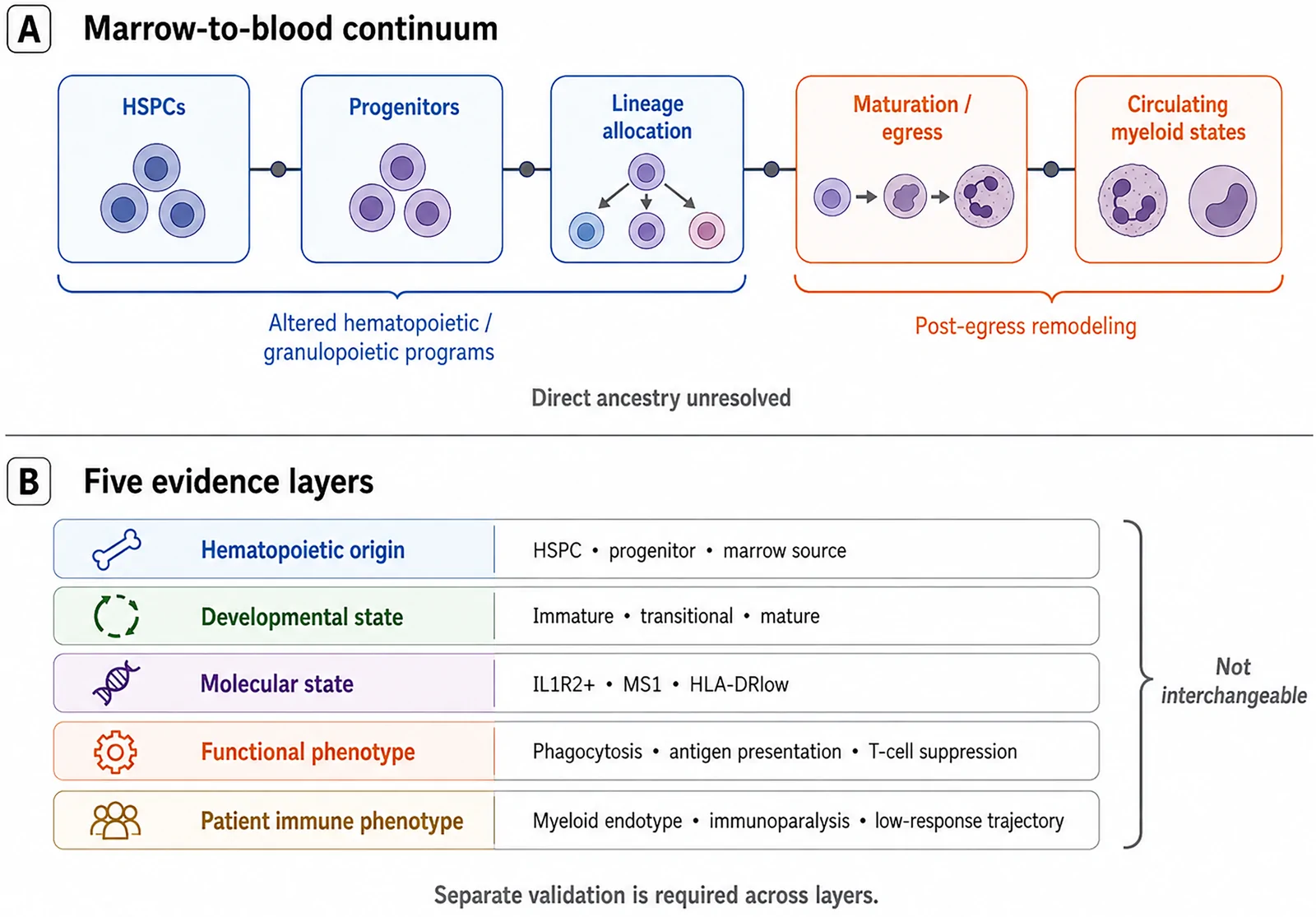
Marrow-to-blood continuum and five evidence layers for interpreting sepsis-associated myeloid dysfunction. **(A)** Uses the marrow-to-blood sequence as an evidence-organizing framework from hematopoietic stem and progenitor cells (HSPCs) through progenitors, lineage allocation, maturation and marrow egress to circulating myeloid states. The blue and orange segments distinguish production-side programs from post-egress remodeling, while the central note emphasizes that direct ancestry from a named human progenitor to a named dysfunctional circulating state remains unresolved. **(B)** Separates hematopoietic origin, developmental state, molecular state, functional phenotype and patient-level immune phenotype. These layers are related but not interchangeable and require separate validation. The figure is an interpretive framework, not a proven continuous lineage model.

## 2 Evidence identification and interpretive approach

We conducted a critical narrative review using PubMed as the primary bibliographic index, with emphasis on human studies published from January 2020 through 12 August 2026. The core search logic was (sepsis OR septic shock) AND (HSPC OR myelopoiesis OR granulopoiesis OR neutrophil OR monocyte OR MDSC) AND (maturation OR differentiation OR trajectory OR reprogramming OR HLA-DR OR MS1 OR IL1R2 OR low-density neutrophil). We included primary studies of clinical sepsis, controlled human systemic endotoxemia, and human ex vivo/*in vitro* mechanistic models when they directly informed one or more of the five evidentiary layers or the source-attribution question. Earlier or disease-adjacent primary studies were retained when they addressed a definitional, kinetic, methodological or mechanistic ambiguity not resolved by contemporary sepsis studies. Review articles were used for field context, terminology and citation discovery rather than as the principal support for mechanistic claims.

Studies were excluded from the core synthesis when they concerned non-myeloid biology without source-attribution relevance, reported only prognostic associations without interpretable myeloid measurements, duplicated a previously reported cohort without additional mechanistic information, or did not provide sufficient methods to determine the sampled compartment or state. Citation tracking and targeted journal searches supplemented the PubMed search. We did not attempt PRISMA-style completeness or a quantitative evidence grade. Instead, key studies were selected for their ability to discriminate developmental from post-egress explanations. For each core study, we extracted population and timing, evidence domain, compartment and sample preparation, assay, state definition, direct function, developmental evidence and major limitations. Clinical sepsis, controlled human systemic endotoxemia and ex vivo/*in vitro* human mechanistic models were interpreted as distinct evidence domains; animal experiments were used only as mechanistic bridges. Computational trajectories were treated as supportive hypotheses rather than lineage evidence. **Table 1** summarizes the key studies and their evidence domains.

**Table 1:** Key human evidence relevant to developmental versus peripheral attribution of sepsis-associated myeloid dysfunction, classified by evidence domain.

Study	Evidence domain	Human population/timing	Compartment and method	Principal finding relevant to source attribution	Interpretive limit
Reyes (5),	Clinical sepsis and ex vivo human	Bacterial sepsis n=29; controls n=36; acute cohorts	Peripheral blood scRNA-seq; bone-marrow induction experiments	Identified reproducible CD14+ MS1 monocyte state with developmental plausibility.	No *in-vivo* marrow-to-MS1 lineage tracing; does not establish MS1 as an M-MDSC lineage.
Reyes (28),	Ex vivo/*in vitro* human	Patient sepsis plasma; assay-specific donor sets	Healthy human CD34+ HSPCs exposed to patient plasma; transcriptomic/CRISPR/functional assays	Septic plasma can instruct progenitors toward suppressive MS1-like myeloid programs *in vitro*.	Mechanistic sufficiency *in vitro*; not direct evidence of dominant patient-marrow mechanism.
Bodinier (37),	Clinical sepsis	Septic ICU patients; discovery n=276, verification n=102	Serial peripheral-blood mHLA-DR	Persistent/decreasing mHLA-DR trajectories identify adverse immune trajectories.	mHLA-DR is an immune-state/function surrogate, not a maturation assay.
Miatello (39),	Clinical sepsis and ex vivo human	Septic shock; 203 analyzable patients	Genotyping, serial mHLA-DR, and ex vivo IFN-gamma context	Host genetic/transcriptional regulation contributes to persistent low HLA-DR.	Supports a non-developmental mechanism for low mHLA-DR; does not define maturation.
Liu (16),	Clinical sepsis	Sepsis n=107; healthy n=80	CD10 maturity stratification and T-cell proliferation assay	Mature CD10+ neutrophils suppressed T cells; immature CD10- cells did not show the same suppressive behavior.	Single cohort/assay context; CD10 may also change with inflammatory exposure.
Kwok (6),	Clinical sepsis	Whole-blood n=39; HSPC n=27	Single-cell multi-omics of whole blood and circulating HSPCs; functional T-cell suppression	HSPC emergency-granulopoiesis programs co-occur with IL1R2+ immature neutrophils and separately identified suppressive neutrophil states.	Supports developmental contribution but not direct ancestry.
Yao (7),	Clinical sepsis with animal mechanistic bridge	Human scRNA: 29 sepsis, 19 healthy	Human PBMC scRNA-seq with murine mechanistic/functional validation	Defines HLA-DR-low/S100A-high human monocyte state.	Direct suppressive-function evidence is chiefly murine; does not constitute direct human functional evidence.
Coudereau (19),	Clinical sepsis	Septic shock n=17; D0/D3/D7	Serial flow cytometry	Immature CD10-low/CD16-low neutrophils and LOX-1+ PMN-MDSC-like cells show distinct kinetics.	Small cohort; phenotype/function distinctions remain important.
Barrios (46),	Clinical sepsis	Acute/late sepsis; small CITE-seq cohorts	CITE-seq, flow cytometry, RNA velocity	Post-septic MDSC-like populations are transcriptionally heterogeneous and temporally remodeled.	RNA velocity is inferential and small cohorts limit generalizability.
Keramati (15),	Controlled human systemic endotoxemia	Human endotoxemia: LPS n=7, placebo n=4; paired marrow/blood subset	Paired bone-marrow/blood scRNA-seq, re-challenge, and IFN-beta rescue	Direct human evidence that systemic inflammation can impair monocyte maturation across marrow and blood.	Controlled endotoxemia is not heterogeneous clinical sepsis.
Charoensappakit (33),	Clinical sepsis	Sepsis n=24; non-sepsis ICU n=10; healthy n=20	Peripheral LDN flow, phagocytosis/apoptosis, and T-cell suppression	LDNs can have acquired suppressive function and mixed maturity.	LDN does not define developmental immaturity or a PMN-MDSC-like phenotype.
Monneret (38),	Clinical sepsis	Septic shock n=1, 023; trajectory subset n=842	Serial standardized mHLA-DR	Large-scale evidence that repeated low mHLA-DR tracks adverse outcomes and ICU-acquired infection.	Clinical state marker; no developmental attribution.
Supino (8),	Clinical sepsis and *in vitro* human	Healthy/non-sepsis/sepsis/septic shock cohorts	High-dimensional flow, transcriptomic integration, and *in vitro* differentiation	IL1R2-high Mono/Mphi state combines macrophage differentiation and immune-dysfunction features.	Demonstrates that maturity and immune competence are separable axes.
De Zuani (58),	Clinical sepsis survivors and ex vivo human	Survivors n=8; acute septic shock n=12; healthy n=10	Peripheral HSPC phenotyping; ex-vivo HSPC-derived macrophages	Suggests persistent HSPC alterations and altered progeny responses after sepsis.	Small cross-sectional survivor cohort; no within-person acute-to-recovery tracking.
Teague (34),	Clinical sepsis and ex vivo human	Sepsis and healthy donors; high-dimensional neutrophil analysis	Flow cytometry, soluble markers, ex-vivo stimulation	LDNs contain diverse maturity/activation states and can be modified through peripheral signaling pathways.	Does not establish a single LDN lineage or direct developmental origin.
Bongers (30),	Controlled human systemic endotoxemia	Healthy volunteers n=15; 3-h LPS challenge	Serial peripheral-blood flow cytometry during endotoxemia	Mature, banded and hypersegmented neutrophil subsets are rapidly mobilized/redistributed during systemic inflammation, constraining temporal interpretation of marrow egress.	Controlled endotoxemia; no paired marrow sampling and not equivalent to clinical sepsis.
Shen (54),	Clinical sepsis (tissue compartment)	Immunosuppressive sepsis; BALF scRNA-seq n=4 plus flow validation cohort	Human BALF single-cell profiling with tissue-compartment validation	Identified lung neutrophil heterogeneity and an inferred CXCR2+ to CD274+IL1RN+ transition, supporting tissue-specific post-egress remodeling.	Small scRNA-seq cohort; trajectory is inferential and causal intervention evidence was not wholly human.

## 3 A marrow-to-blood framework for interpreting myeloid dysfunction

### 3.1 What the continuum means - and what it does not mean

Here, the marrow-to-blood continuum identifies the points at which a dysfunctional state could be specified: HSPC activation, lineage allocation, maturation, marrow egress and subsequent state acquisition in blood. It is an analytical sequence, not a lineage map. Human sepsis studies can detect altered HSPC programs and expanded immature-neutrophil states within the same adverse response endotype, but that co-occurrence does not establish ancestry (6). Paired marrow-blood studies provide stronger evidence about the origin of a circulating state; a particularly informative example comes from controlled systemic inflammation rather than heterogeneous clinical sepsis (15).

All circulating neutrophils and monocytes ultimately arise from hematopoiesis. The relevant question is when, and at which biological level, the dysfunctional program is acquired. A phenotype may be substantially specified before egress, imposed mainly by the peripheral septic environment, or assembled across both compartments. The framework therefore focuses on the timing and biological level of state specification rather than on the anatomical source of the cell itself.

### 3.2 Five non-interchangeable levels of evidence

We treated five levels as distinct operational evidence categories. Hematopoietic origin requires direct measurement of progenitors or hematopoietic output; developmental state requires validated maturation information from surface phenotype, morphology or a coherent differentiation trajectory; molecular state requires a defined transcriptomic, epigenetic, proteomic or surface program; functional phenotype requires a direct assay of the proposed function in the same or prospectively matched cell population; and patient immune phenotype requires integration of cellular states with a reproducible patient-level trajectory or endotype (3). Movement between levels is supported only when measurements are linked within the same cells, prospectively defined population, paired compartment or perturbation experiment. Co-occurrence across a cohort is not sufficient to convert one level into another.

CD10 illustrates why a developmental marker cannot carry a functional inference by itself. In sepsis, mature CD10-positive neutrophils suppressed T-cell proliferation more strongly than CD10-negative cells (16), consistent with an earlier G-CSF donor study in which CD10-positive and CD10-negative neutrophils had opposite effects on T cells (17). However, E. coli or LPS challenge can transiently reduce neutrophil CD10 surface expression (18), so low CD10 during systemic inflammation is not by itself proof of developmental immaturity. In septic shock, CD10-low/CD16-low immature neutrophils and LOX-1-positive PMN-MDSC-like cells also followed distinct kinetics (19). Cancer studies independently show both maturation-stage heterogeneity and acquisition of suppressive function by mature granulocytes in a tissue milieu (20, 21). CD10 is therefore most informative as part of a multiparametric maturity definition linked to direct function, not as a standalone developmental clock.

MDSC nomenclature requires particular care. Current consensus treats ontogeny, surface phenotype and suppressive function as related but non-identical dimensions, with inhibitory activity central to a defensible MDSC designation (22). In this review, the MDSC label is reserved for populations with an appropriate myeloid phenotype and direct suppressive-function evidence; phenotype-only or transcriptome-only populations are described as MDSC-like.

### 3.3 What counts as developmental evidence?

Developmental labels alone are insufficient for developmental attribution. In this review, a developmental contribution is considered supported when progenitor or marrow abnormalities are linked to a downstream state by paired compartments, serial timing, concordant programs or a perturbation showing that progenitor exposure is sufficient to generate the state. We reserve developmental programming for the stronger situation in which an upstream HSPC/progenitor exposure produces a molecular or functional alteration that persists into downstream progeny under defined conditions; direct human ancestry would strengthen this inference further. Where persistence or inheritance has not been shown, we use developmental contribution or developmental remodeling. Immature markers and computational trajectories alone do not meet this threshold.

The strongest human designs combine compartment, time, developmental markers, molecular programs and function. Paired marrow-blood measurements constrain origin, serial sampling constrains timing, and direct function constrains biological meaning; none substitutes for lineage tracing. **Figure 2** translates these requirements into a sampling-profiling-validation roadmap without assigning a formal evidence grade.

**Figure 2:**
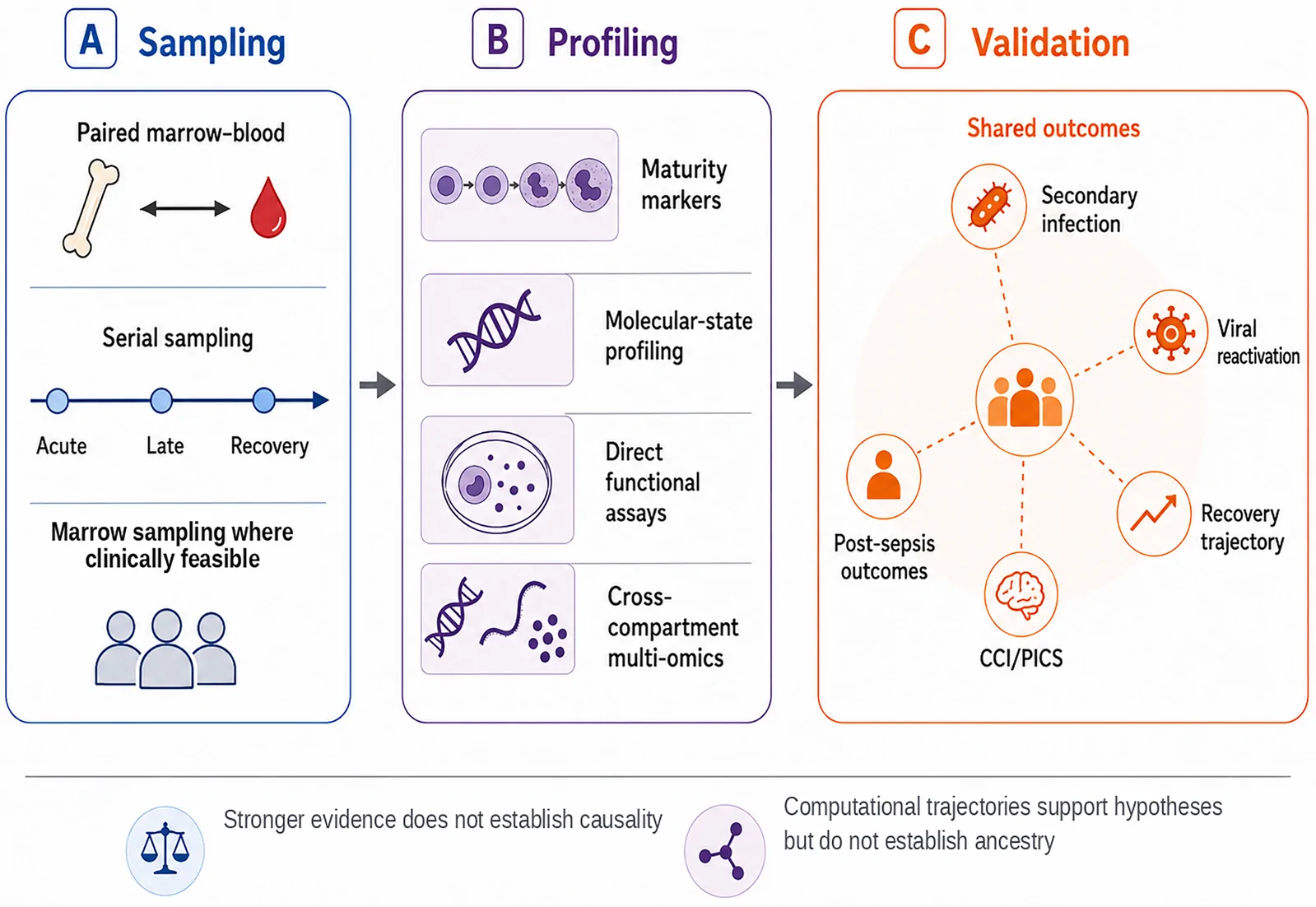
Research roadmap for credible marrow-to-blood attribution in human sepsis. **(A)** Emphasizes paired marrow-blood sampling, serial sampling across acute, late and recovery phases, and marrow sampling in selected clinically and ethically feasible subsets. **(B)** Combines maturity markers, molecular-state profiling, direct functional assays and cross-compartment multi-omics. **(C)** Links these measurements to prospective mechanism-relevant outcomes, including secondary infection, viral reactivation, recovery trajectory, chronic critical illness/persistent inflammation, immunosuppression and catabolism syndrome (CCI/PICS), and post-sepsis outcomes. The design is intended to test falsifiable source-attribution hypotheses; computational trajectories can strengthen developmental hypotheses but do not establish ancestry.

### 3.4 Cell state is not a patient endotype

Cell state and patient endotype also operate at different scales. Multicohort transcriptomic studies identify reproducible patient-level immune structures in which myeloid programs, including immature-neutrophil biology, contribute alongside lymphoid and systemic signals (3, 23). Infection source can modify the circulating host response, and high-dimensional phenotyping likewise shows substantial peripheral immune heterogeneity (24, 25). Age and anatomical site further alter immune-state composition (26). A single myeloid state may contribute strongly to an endotype without defining the patient’s complete immune condition.

## 4 The hematopoietic source under septic stress

### 4.1 Emergency myelopoiesis: adaptive demand response and potential maladaptation

Emergency myelopoiesis is a demand-adapted response to increased innate immune-cell requirements, and its broader regulatory biology has been reviewed elsewhere (27). For the present source-attribution question, the relevant human observation is that sepsis can alter HSPC/granulopoietic transcriptional and chromatin programs while immature and mature neutrophil states are simultaneously remodeled in blood. The functional consequence depends on the intensity and duration of these signals: accelerated output may support host defense, whereas sustained septic stress can broaden developmental state distributions and coexist with post-egress regulatory change. Cell counts alone cannot distinguish adaptive emergency production from maladaptive myeloid remodeling.

### 4.2 Human sepsis supports a developmental contribution

Kwok and colleagues provide one of the clearest clinical-sepsis examples supporting a developmental contribution. Single-cell multi-omic profiling of whole blood and circulating HSPCs identified emergency-granulopoiesis-related transcriptional and epigenetic programs alongside IL1R2-positive immature neutrophils and separately identified suppressive mature neutrophil states (6). The adverse response endotype was enriched for these features, and sepsis neutrophils directly inhibited CD4 T-cell proliferation and activation. Because hematopoietic programs and peripheral functional states were measured in the same disease context, the study provides stronger evidence for developmental involvement than an isolated immature-cell count. Direct ancestry, however, was not measured. The data support a developmental contribution without establishing a continuous HSPC-to-suppressive-neutrophil lineage in humans.

### 4.3 Systemic signals can program progenitors, but clinical sepsis remains incompletely sampled

Patient-plasma experiments address a different part of the question: whether the septic systemic environment is sufficient to alter progenitor fate. Plasma from patients with bacterial sepsis induced myelopoiesis and MS1-like suppressive myeloid programs when applied to healthy human CD34-positive HSPCs *in vitro* (28). IL-6- and IL-10-related signaling contributed to this induction, placing cytokine instruction upstream of at least part of the progenitor response. This experiment provides a mechanistic bridge between the soluble septic milieu and altered myeloid production, but it is intentionally simplified. It establishes biological sufficiency in human progenitors, not the relative contribution of IL-6/IL-10 signaling within patient marrow or the extent of subsequent remodeling in the circulation.

Controlled endotoxemia provides unusually direct cross-compartment human evidence. Serial marrow and peripheral-blood sampling showed that the later low-response state was accompanied by loss of intermediate/non-classical monocyte maturation programs, attenuated type I interferon signaling and reduced responsiveness to a second inflammatory challenge (15). IFN-beta restored elements of the secondary response and promoted monocyte maturation in the experimental setting, linking IFN-I activity to differentiation state as well as functional competence. The anatomical and temporal resolution is unusual, but the model is time-limited, pathogen-free and lacks the organ failure, antimicrobial therapy, comorbidity and variable disease duration of clinical sepsis. It demonstrates that controlled systemic inflammation can perturb human myelopoiesis, but does not establish that the same process occurs uniformly in clinical sepsis. Key human studies that directly inform developmental versus peripheral attribution are summarized in **Table 1**.

### 4.4 Marrow transit and egress impose a temporal constraint

Neutrophil kinetics constrain source attribution. In healthy adults, stable-isotope modeling estimated a postmitotic marrow transit of approximately 5.8 days and a blood half-life of approximately 18–19 hours (29); these are reference values, not fixed constants in sepsis. Experimental human endotoxemia further shows that mature, banded and hypersegmented neutrophil subsets can be redistributed or mobilized within hours of systemic inflammatory stimulation (30). A blood phenotype that appears rapidly after a new exposure therefore cannot be attributed automatically to *de novo* progenitor differentiation: release of pre-existing marrow reserves, altered trafficking and peripheral state acquisition are competing explanations. Conversely, delayed or recurrent waves aligned with immature-cell release are more compatible with a production-side contribution. Longitudinal models should explicitly account for the delay between development, marrow egress and blood sampling.

## 5 Lineage-specific developmental distortion

### 5.1 Neutrophils: maturity, density and suppressive function do not form one axis

Neutrophil dysfunction does not map onto a single maturity-to-suppression axis. IL1R2-positive immature states and CD121b-positive/CD10-negative populations support clinically relevant developmental heterogeneity (6) (31), but function cannot be inferred from immaturity. Human sepsis data also demonstrate direct T-cell suppression by mature CD10-positive neutrophils (16), and CD10 itself can be modified by inflammatory exposure as noted above. Maturity should therefore be defined multiparametrically and linked to direct function when a suppressive designation is claimed.

Low-density neutrophils illustrate a related problem from a different experimental perspective. Density is a biophysical separation phenotype, not a developmental lineage. Transcriptomic work comparing high- and low-density granulocytes in sepsis found precursor-enriched differentiation programs in low-density fractions, consistent with a developmental contribution (32). Functional and high-dimensional studies, however, show that low-density fractions also contain activated or degranulated cells with mixed maturity, defective antimicrobial functions and, in selected cohorts, PD-L1-associated T-cell suppression (33, 34). Peripheral activation itself can shift density. Low-density status therefore reflects a mixture of production history and acquired state whose relative contributions need to be measured rather than assumed.

LOX-1-positive PMN-MDSC-like cells reinforce the distinction between developmental annotation and functional identity. In longitudinal septic shock, CD10-low/CD16-low immature neutrophils and LOX-1-positive PMN-MDSC-like cells showed different temporal dynamics (19). The populations can overlap without being developmentally interchangeable. When lymphocyte suppression has not been tested, a PMN-MDSC-like phenotype is the more defensible description.

Neutrophil heterogeneity also includes molecular specialization that may be only loosely related to developmental age. OLFM4-defined subsets, for example, show distinct proteomic profiles in health and septic shock without a correspondingly simple functional interpretation (35). Whole-blood single-cell studies, PBMC/LDN preparations and conventional granulocyte isolations sample different parts of this landscape. Assigning developmental meaning to a platform-specific state therefore requires reconciliation of density, maturity, activation and direct function; otherwise technical enrichment can be mistaken for lineage structure.

### 5.2 Monocytes: developmental plausibility coexists with acquired low-response states

Monocyte data reveal a similar tension between developmental plausibility and acquired peripheral regulation. MS1 is a reproducible CD14-positive sepsis-associated state whose program can be induced in a human bone-marrow/progenitor context (5). Patient-plasma/HSPC experiments subsequently showed that a related program can be instructed during progenitor differentiation (28). High-dimensional mass cytometry, however, demonstrates broad peripheral monocyte remodeling in sepsis without identifying where those states were specified (25). MS1 is therefore best regarded as a molecular state with developmental support, not as a proven *in-vivo* M-MDSC lineage. Its occurrence in other severe inflammatory states also suggests a host-response program that is not unique to bacterial sepsis.

HLA-DR-low/S100A-high monocytes provide another example. Human single-cell data identify a reproducible low-antigen-presentation, S100A-high state in sepsis, while direct suppressive-function evidence in the corresponding study is derived substantially from murine experiments (7). The human molecular annotation and the animal functional validation are complementary, but they support different levels of inference and should be reported as such. The available evidence supports a human HLA-DR-low/S100A-high molecular state with cross-species mechanistic support; it does not directly demonstrate *in-vivo* T-cell suppression by the human population.

IL1R2 biology further complicates a simple immature-equals-suppressive model. In this review, IL1R2 denotes transcript-defined annotation, whereas IL-1R2/CD121b refers to protein-level measurement when reported by flow cytometry. IL-1R2 functions as a non-signaling decoy receptor that can dampen IL-1 activity, and high-dimensional human data identified an IL1R2-high circulating monocyte/macrophage-like population in sepsis with low HLA-DR and immune-dysfunction features alongside markers of mature macrophage differentiation (8). Healthy-donor monocytes acquired related features after CSF and LPS stimulation *in vitro*. The same cell state can therefore combine evidence of differentiation with an anti-inflammatory or low-response molecular program. Developmental maturity and immune competence are not opposing ends of one axis; they are separate properties shaped by different signals.

Monocyte HLA-DR (mHLA-DR) makes this distinction clinically tangible. Serial studies show that delayed recovery or decline in mHLA-DR is associated with secondary infection and death, and large contemporary cohorts confirm the importance of repeated measurement rather than a single value (36–38). The assay reflects antigen-presentation competence, not developmental age. Class II transactivator (CIITA) promoter variation can influence persistent HLA-DR depression in septic shock (39), showing that low HLA-DR can arise through transcriptional regulation independently of developmental maturity.

### 5.3 MDSC-like states: separating phenotype, function, and ontogeny

For MDSCs, the main problem is identity rather than a distinct developmental lineage. Foundational human sepsis studies established that expanded myeloid populations can directly suppress T-cell responses and persist beyond the acute episode (40, 41). More recent cohorts document phenotypically defined monocytic and polymorphonuclear MDSC-like populations during acute and late sepsis, with persistence in some survivors (42–44). Single-cell and CITE-seq studies add substantial transcriptional heterogeneity within these gates (45, 46). The combined evidence supports clinically relevant suppressor biology, but also shows why phenotype, ontogeny and function cannot be treated as one variable.

Clinical associations are not uniformly adverse. In a selected pneumonia-sepsis cohort with multiorgan failure, higher monocytic MDSC-like levels were associated with a more favorable outcome (47). This finding is not inconsistent with suppressive biology; it indicates that subtype, timing, disease context and compensatory regulation may alter clinical meaning. A population that limits damaging inflammation early may differ clinically from a persistent suppressive population during later infection susceptibility. A cross-sectional percentage cannot distinguish those roles.

**Table 2** separates phenotypic MDSC-like states from directly function-tested suppressor populations. This preserves population-dynamics data without assigning unmeasured function. The distinction is equally important for analysis: unsupervised or semi-automated flow-cytometric clustering can improve reproducibility, but a cluster should be interpreted as an MDSC only when its operational phenotype and suppressive function are adequately supported (22). Developmental labels such as ‘immature’ describe maturation, not functional suppression.

**Table 2:** Recurrent myeloid states in sepsis, their representative operational definitions, and the minimum evidence required for functional designation.

State/label	Representative markers/sample type	Measured evidentiary layer	Direct functional evidence	Minimum evidence for a functional designation	Principal interpretive limitation
IL1R2+ immature neutrophil	IL1R2 transcript/immature-neutrophil signature; whole-blood single-cell data	Developmental and molecular state	Suppressive sepsis neutrophils shown in the associated study, but not restricted to the IL1R2+ subset	Direct function in the same prospectively defined/sorted IL1R2+ population	Not all suppressive neutrophils; not a proven lineage
CD10+ vs CD10- neutrophils	CD10 with multiparameter maturity context; isolated/whole-blood neutrophils	Phenotypic maturity	Direct T-cell proliferation assays in human studies	Function tested within CD10-defined strata plus corroborating maturity markers	CD10 can change with inflammation; CD10-low is not automatically immature or suppressive
Low-density neutrophil	Density fraction plus CD15/CD66b and related markers; PBMC/LDN fraction	Biophysical fraction and phenotype	Phagocytosis, apoptosis and T-cell suppression demonstrated in selected human studies	Purified population with assay conditions that exclude density-gradient/activation artefact	Not a developmental lineage; not synonymous with PMN-MDSC
LOX-1+ PMN-MDSC-like cell	LOX-1 with granulocytic phenotype; peripheral blood flow cytometry	Phenotypic state	Variable across studies	Direct suppressive function in the same LOX-1-defined population before using PMN-MDSC without ‘-like’	Not all immature neutrophils
MS1 monocyte	MS1 transcriptomic/surface program; PBMC or single-cell data	Molecular state	Functional/epigenomic characterization; progenitor induction in later experiments	Direct function plus patient *in-vivo* linkage if an M-MDSC designation is intended	Developmental plausibility does not establish *in-vivo* M-MDSC lineage
HLA-DR-low/S100A-high monocyte	Low HLA-DRA/class II with S100A8/A9-high program; PBMC scRNA-seq	Molecular state	Strong mechanistic support includes murine functional validation	Direct human functional assay in the corresponding defined population	Does not establish direct human T-cell suppression without direct testing
Low mHLA-DR	Quantitative monocyte HLA-DR; standardized whole-blood flow cytometry	Functional-state/antigen-presentation readout	Clinical trajectory and immune-competence surrogate evidence	Additional direct function if a specific cellular deficit is claimed	Does not measure developmental maturity; not synonymous with M-MDSC or marrow failure
IL1R2-high Mono/Mphi	IL1R2-high, low HLA-DR, macrophage-like features; high-dimensional blood profiling	Molecular/surface state	Immune-dysfunction features and *in-vitro* induced states characterized	Direct functional assay in the defined human population	Mature differentiation features can coexist with low-response state
Phenotypic M-/PMN-MDSC-like populations	Consensus-compatible monocytic or granulocytic flow gates; peripheral blood/PBMC	Phenotypic state	Variable; absent in some cohorts	Appropriate phenotype plus direct suppressive function in the same defined population for MDSC designation	Not a stable lineage and not functionally proven by phenotype alone

### 5.4 Methodological sources of apparent myeloid heterogeneity

Platform and pre-analytic choices can create or enrich different myeloid populations. Standard PBMC/Ficoll workflows largely exclude conventional high-density neutrophils while retaining LDNs, whereas whole-blood single-cell approaches sample granulocytes more broadly. Neutrophils are also susceptible to activation by collection, storage, red-cell lysis and isolation procedures; direct comparisons show that isolation method alters surface phenotype and responsiveness (48). In PMN-MDSC work, density-gradient devices and assay configuration can even generate apparent neutrophil-mediated T-cell suppression through technical artefact (49).

Flow-cytometric gating, viability and doublet exclusion, marker thresholds, and whether function is tested in the same sorted population further affect comparability. Clinical timing is equally important because time from sepsis onset and major treatments can change circulating composition and cell state. Studies intended for cross-cohort mechanistic comparison should therefore report biological compartment, sample preparation/isolation, processing delay, gating and marker definitions, sampling time relative to sepsis onset and major treatment exposures, and the exact functional assay. Differences between studies should not be assigned to developmental biology until these sources of variation have been considered.

## 6 Peripheral reprogramming after marrow egress

### 6.1 Monocyte tolerance and epigenetic remodeling

Peripheral reprogramming remains independently well supported. Human monocytes can acquire durable low-response programs after inflammatory exposure through signaling and epigenetic mechanisms. In sepsis, monocyte DNA-methylation abnormalities correlate with inflammatory cytokines and organ dysfunction, and Toll-like receptor stimulation can reproduce part of that methylation landscape *in vitro* (50). Subsequent work linked early gram-negative endotoxin exposure to JAK2-STAT signaling and TET2-associated regulation of tolerized genes (51). These studies define a molecular route by which a substantially mature monocyte can acquire a persistent low-response program after exposure and help explain why the phenotype may outlast the initiating cytokine surge without having been specified during hematopoiesis.

### 6.2 Metabolic and soluble-milieu instruction

Soluble and metabolic signals provide another route to post-egress state acquisition. Large-scale plasma proteomics shows extensive, time-varying changes in host-response modules across sepsis phenotypes (52). Metabolomic studies in septic shock likewise identify broad changes in carbohydrate, amino-acid, lipid and related metabolites, and selected metabolites can alter LPS responses when applied to healthy PBMCs ex vivo (53). These experiments do not identify the origin of a patient’s myeloid state, but they establish that the extracellular environment can modify the responsiveness of mature circulating cells. Repeated peripheral instruction could therefore sustain a blood phenotype that resembles ‘programming’ even when hematopoietic output is unchanged.

### 6.3 Neutrophil activation, degranulation and density shifts

Neutrophils offer particularly direct evidence of post-egress plasticity. Low-density phenotypes can emerge with peripheral activation and degranulation, while suppressive function in selected LDN preparations can be attenuated by PD-L1 blockade; high-dimensional work also implicates pathways such as SYK in sepsis LDN biology (33, 34). These mechanistic and ex-vivo observations are not treatment evidence, but they show that important features of a dysfunctional neutrophil state can be generated or modified after egress. A mature cell can acquire altered trafficking, degranulation, oxidative or suppressive programs without reverting to an immature developmental state.

Developmental and peripheral effects can occur simultaneously. Sepsis may change which neutrophils are produced and also reshape their behavior after they enter blood. Because the two processes can converge on similar transcriptional or surface phenotypes, phenotype-only studies cannot allocate causality between compartments.

### 6.4 Tissue compartments are a second post-egress environment

Blood is only one post-egress compartment. After recruitment, myeloid cells encounter endothelial, metabolic, microbial and tissue-specific cytokine signals that can produce states not represented faithfully in peripheral blood. Human bronchoalveolar-lavage single-cell profiling in immunosuppressive sepsis identified distinct neutrophil populations and an inferred state transition within the lung compartment (54). This supports tissue-specific myeloid remodeling but does not establish marrow ancestry, and the causal intervention evidence in that study was not wholly human. Paired blood-tissue studies are therefore needed to determine whether a circulating state is retained, further reprogrammed or replaced after tissue entry.

## 7 Persistence across late sepsis and recovery

### 7.1 Acute and early longitudinal dynamics

Longitudinal sampling introduces a different problem: detecting the same marker on day 1 and day 14 does not mean that the same cells or mechanism persisted. Septic-shock cohorts identify distinct mHLA-DR trajectories over the first week, with delayed or declining recovery associated with secondary infection or death (36–38). Multidimensional studies further show that mHLA-DR, stimulated cytokine capacity and lymphocyte measures recover at different rates (55). Whole-blood transcriptomics likewise shows that host-response programs evolve across acute illness and recovery rather than following one invariant sequence (56). The familiar shorthand of ‘hyperinflammation followed by immunosuppression’ therefore fits poorly when inflammatory and low-response programs coexist or diverge across compartments.

Because blood neutrophils turn over on the scale of less than one day in healthy adults (29), repeated detection of a neutrophil surface phenotype days later does not imply persistence of the same cells. It may reflect continued production of phenotypically similar new cells, repeated peripheral induction, altered trafficking, prolonged survival in a disease-specific context, or a combination of these processes. A longitudinal blood trajectory is therefore necessary but not sufficient evidence for persistent hematopoietic programming; locating biological memory requires evidence about the compartment in which the relevant program is maintained.

### 7.2 Chronic critical illness and persistent inflammation, immunosuppression, and catabolism syndrome

Late sepsis and chronic critical illness are accompanied by continued remodeling of the peripheral myeloid compartment. Pilot single-cell and later CITE-seq studies identify persistent but heterogeneous MDSC-like states, with transcriptomic trajectories differing across recovery patterns (45, 46). PICS-focused single-cell work likewise suggests that monocyte states in post-sepsis PICS are not static copies of the acute-sepsis state: some functions appear partly restored while other immune abnormalities persist (57). Recovery is therefore better represented as an evolving trajectory than as persistence of a single suppressor-cell population.

### 7.3 Does sepsis leave a durable HSPC imprint in humans?

The central recovery question is whether sepsis leaves a durable imprint at the HSPC level. A 2026 human study compared eight sepsis survivors with acute septic-shock patients and healthy donors and reported altered HSPC compartments together with abnormal metabolic and transcriptional responses in macrophages differentiated from survivor HSPCs ex vivo (58). This is notable because the signal persisted in progeny generated outside the original septic environment, which reduces - although does not eliminate - the likelihood that the finding reflects only an acute plasma effect.

These findings remain preliminary. The survivor cohort is small and cross-sectional, and the same individuals were not followed from acute illness through recovery. The study cannot establish how common persistent HSPC reprogramming is, whether it originated during the index septic episode, or whether it contributes materially to post-sepsis morbidity. At present, durable hematopoietic imprinting is biologically plausible in humans and warrants prospective testing; it is not an established mechanism of post-sepsis syndrome.

### 7.4 What current longitudinal evidence still cannot tell us

The same interpretive separation is needed in recovery studies. Persistent phenotypic MDSC-like expansion does not establish persistent marrow programming. Chromatin or metabolic abnormalities in circulating cells do not reveal whether the relevant memory resides in HSPCs, repeatedly generated progeny, or continuously re-exposed peripheral populations. Conversely, an HSPC abnormality does not show that it is functionally responsible for the circulating state measured months later.

A convincing post-sepsis origin model will require within-person longitudinal studies linking early hematopoietic state, later peripheral function and clinically relevant outcomes. For now, the evidence supports persistent immune dysfunction more strongly than any single persistent developmental mechanism.

## 8 Translational implications

### 8.1 What should be measured?

Source attribution also matters for immune monitoring. A bedside assay need not identify developmental origin to be clinically useful, but its mechanistic scope should remain explicit. mHLA-DR is among the most mature sepsis immune-state readouts and has been developed for trial enrichment (38, 59, 60). Pre-analytic handling and assay-specific thresholds matter, underscoring that even a standardized functional-state biomarker is not a universal maturity measure. Studies concerned with maturation should pair mHLA-DR with developmental markers and, where feasible, direct function or cross-compartment data.

### 8.2 Who should be treated?

Precision immunotherapy studies increasingly use immune phenotypes to enrich treatment populations. The PROVIDE trial established the feasibility of biomarker-guided immunotherapy in sepsis, while the more recent ImmunoSep randomized trial provides contemporary evidence that phenotype-guided immunomodulation can improve an organ-dysfunction endpoint in selected patients (26, 61). These trials validate the clinical relevance of state-aware selection; they do not validate a marrow-developmental mechanism. A low-response phenotype can arise through reduced or altered production, peripheral tolerance, or several processes at once. The more specific the proposed therapeutic mechanism, the more specifically the companion biomarker must interrogate that biological layer.

### 8.3 Which biological mechanism is being targeted?

Therapeutic selection becomes interpretable only when the intervention is aligned with the biological defect being targeted. If a patient’s dominant abnormality lies in hematopoietic production, an intervention directed only at peripheral checkpoint signaling may leave that process unchanged. If the dominant abnormality is a reversible mature-cell state, stimulating hematopoiesis may increase cell number without restoring function. Current clinical evidence cannot assign patients reliably to these categories. Mechanism-directed trials therefore need pharmacodynamic measurements at the biological level the intervention is intended to modify, rather than another unvalidated composite score.

## 9 Discussion: developmental contribution, peripheral reprogramming, or both?

### 9.1 Evidence favoring a developmental contribution

Evidence for a developmental contribution comes from four convergent observations: sepsis alters HSPC/granulopoietic programs; developmentally immature neutrophil states expand within adverse response endotypes; patient plasma can instruct human progenitors toward suppressive programs *in vitro*; and controlled systemic inflammation can perturb marrow-to-blood monocyte maturation in humans (6, 15, 28). Together, these findings are difficult to reconcile with a model in which hematopoietic development is unaffected. They support developmentally acquired information as one component of the final blood phenotype without establishing its quantitative contribution in clinical sepsis.

The limitation is that this evidence is relational rather than genealogical. In clinical sepsis, HSPC and downstream states are profiled in the same disease setting but are not clonally linked. In experimental endotoxemia, marrow and blood are connected anatomically and temporally, but the model lacks the complexity of clinical sepsis. In the plasma-HSPC experiment, causality is cleaner but the system is ex vivo. These studies converge on a developmental contribution while retaining different limitations that preclude a claim of proven human lineage.

### 9.2 Evidence favoring post-egress state acquisition

Evidence for post-egress state acquisition is direct across several settings. In sepsis, endotoxin-tolerance and epigenetic-remodeling studies, LDN activation/degranulation studies, mature-neutrophil suppression, and transcriptional regulation of mHLA-DR all show that function or molecular state can change without a simple shift in developmental age (16, 33, 34, 39, 50, 51). Outside sepsis, morphologically mature human neutrophils can acquire reversible T-cell suppressive function after exposure to ovarian-cancer ascites (21). This disease-adjacent evidence does not substitute for clinical sepsis data, but it provides a direct proof-of-principle that a tissue milieu can impose suppressive function on mature neutrophils. An exclusively pre-specified-at-egress model is therefore difficult to sustain.

### 9.3 A proposed hybrid model

The available evidence is compatible with a hybrid model, although that model remains provisional (**Figure 3**). Inflammatory signals may reshape HSPC and progenitor output, generating progeny whose developmental programs have already been altered; after egress, cytokine, metabolic, microbial and treatment-modified environments can further modify molecular and functional state. The observed blood phenotype would then reflect both histories. This interpretation accommodates discordance among maturity, density, single-cell cluster and suppressive function without requiring every discordance to share the same origin.

**Figure 3:**
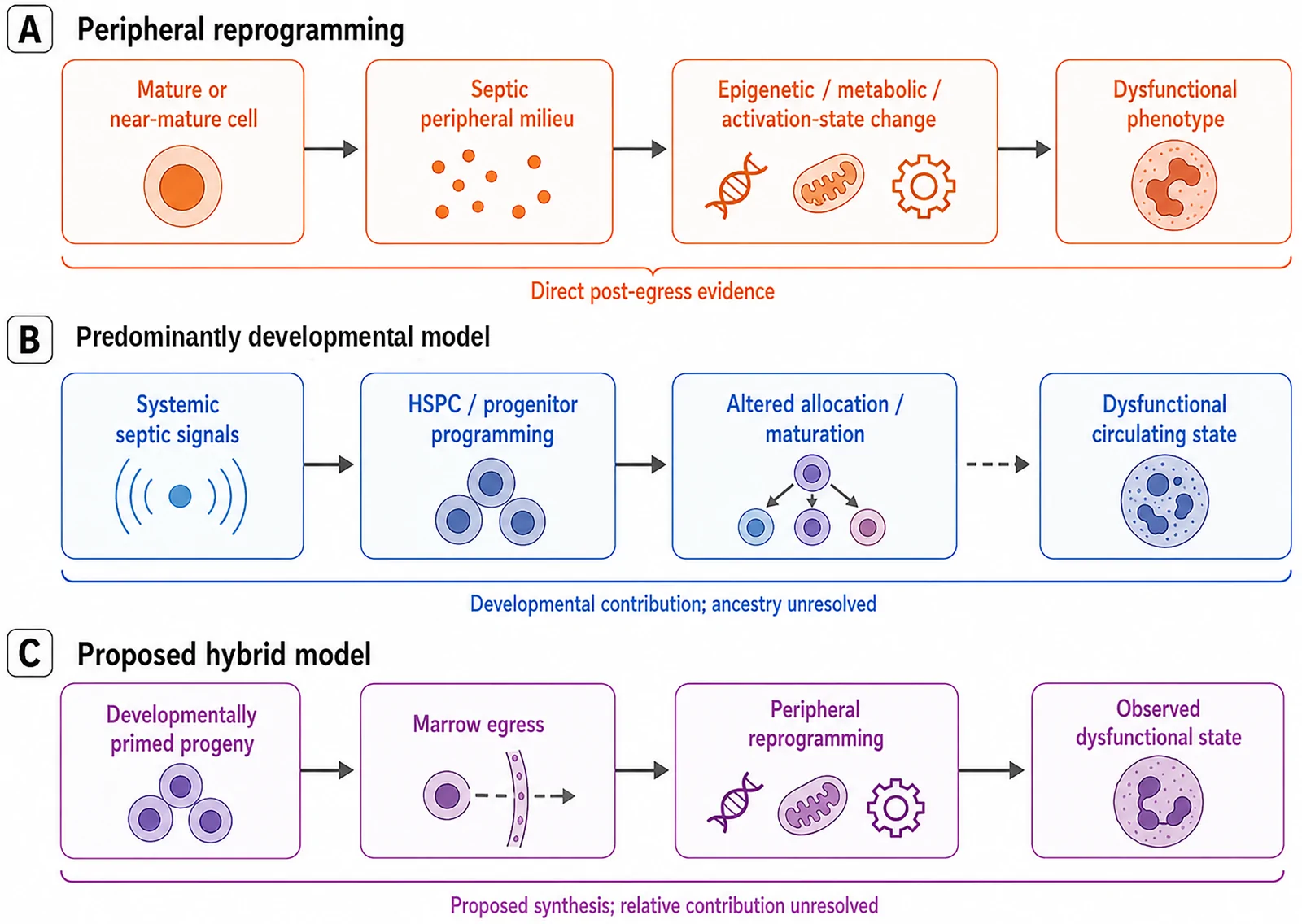
Competing models for the origin of dysfunctional myeloid states in sepsis. **(A)** Shows a peripheral-reprogramming model in which mature or near-mature circulating cells acquire epigenetic, metabolic or activation-state changes after exposure to the septic milieu. **(B)** Shows a predominantly developmental (strong-form) model in which systemic signals alter HSPC/progenitor programs and lineage allocation or maturation before marrow egress; the final link to a named dysfunctional circulating state remains inferential because current human evidence supports a developmental contribution but does not establish direct ancestry. **(C)** Presents a proposed hybrid model in which developmental remodeling and post-egress reprogramming interact. The hybrid model is a testable conceptual synthesis rather than a demonstrated two-hit mechanism.

The hybrid model is useful only if it yields discriminating predictions. A predominantly developmental state should show marrow-blood concordance, appear in downstream progeny with kinetics compatible with maturation and egress, persist after removal from septic plasma when progeny are generated outside the original milieu, and retain a progenitor-linked chromatin program. A predominantly peripheral state should be inducible in mature healthy cells, emerge faster than *de novo* developmental transit would predict, weaken after washout or pathway blockade, and show limited concordance with upstream progenitors. Reversibility, persistence and temporal lag therefore become mechanistic tests rather than descriptive features. Existing HSPC-plasma, endotoxemia, LDN and monocyte-tolerance studies satisfy parts of these predictions (15, 28, 33, 51), while induction of suppressive mature neutrophils in a human tumor milieu provides an additional peripheral-programming proof-of-principle (21). No current human sepsis study tests all predictions within the same lineage and longitudinal design.

**Table 3** converts these predictions into falsifiable source-attribution hypotheses. The empirical objective is not to declare a universal two-hit mechanism, but to determine for a defined cell state and time whether the dominant information was acquired before egress, after egress, or across both compartments.

**Table 3:** Falsifiable source-attribution hypotheses and study designs capable of discriminating developmental from post-egress mechanisms.

Falsifiable source-attribution hypothesis	Predicted observations if supported	Observations arguing against the hypothesis	Critical design/assay	Mechanism-linked outcomes
Suppressive neutrophil state is predominantly developmentally specified	Marrow-blood concordance; progenitor-linked chromatin program; downstream state appears with maturation/egress-compatible delay; phenotype persists in newly generated progeny outside septic plasma	Rapid induction in mature healthy neutrophils; weak upstream concordance; loss after washout without changing progenitor output	Paired marrow-blood serial multi-omics, sorted-cell function, and ex vivo progeny assay	Secondary infection, pathogen clearance, immune recovery
Suppressive neutrophil state is predominantly peripherally acquired	Inducible in mature healthy cells; rapid onset; reversibility after washout/pathway blockade; limited progenitor concordance	State is present before egress and retained in daughter cells despite removal from the peripheral milieu	Mature-cell exposure/washout experiments, serial blood profiling, and selected paired-marrow sampling	Secondary infection, adaptive-immune recovery
MS1 is predominantly progenitor-programmed	Patient marrow/HSPC program precedes or parallels blood MS1; patient-derived progeny retain MS1-like features outside septic plasma	MS1 appears without corresponding progenitor signal and is readily induced/reversed in mature monocytes	Matched HSPC-monocyte profiling, plasma-transfer/withdrawal, and direct function	Immune recovery, secondary infection, chronic critical illness
Persistent low mHLA-DR is mainly production-side rather than peripheral	Low-HLA-DR state is detectable in newly generated monocytes with concordant developmental markers and persists outside acute plasma exposure	Mature monocytes downregulate/recover HLA-DR rapidly without a corresponding marrow change	mHLA-DR, maturity markers, direct function, and selected cross-compartment sampling	ICU-acquired infection, recovery trajectory
Sepsis leaves a durable HSPC imprint in a clinically relevant subset	Within-person HSPC abnormalities persist into recovery and predict altered function in ex-vivo progeny after removal from the septic environment	HSPC signals normalize while peripheral dysfunction persists, or survivor findings fail longitudinal replication	Prospective acute-to-recovery HSPC cohort with nested marrow sampling and progeny assays	Post-sepsis infection, readmission, functional recovery
A blood myeloid state is further reprogrammed after tissue entry	Paired blood-tissue cells show compartment-specific molecular/function changes not explained by blood composition alone	Tissue state closely mirrors blood state without additional tissue-specific program	Paired blood-tissue single-cell/multi-omic profiling with direct function where feasible	Organ-specific dysfunction, secondary infection, tissue recovery

### 9.4 Why discordance between state, function and endotype matters

At the patient level, the same separation between biological layers remains necessary. Multicohort studies organize sepsis into reproducible immune or transcriptomic endotypes, but these structures integrate multiple cell populations and systemic signals rather than a single maturation program (3, 23). Infection source, age and anatomical site alter the circulating host response and the prevalence of individual immune states (24, 26). A myeloid state can be mechanistically important within an endotype without defining the endotype or acting as its sole cause.

## 10 What evidence would resolve the marrow-to-blood question?

### 10.1 Paired marrow-blood sampling

**Table 3** and **Figure 2** summarize the unresolved attribution questions and the study designs most likely to address them. An informative design would sample the production and circulating compartments in the same patients over time and directly test the function of the states being compared. Routine repeated marrow aspiration is neither ethical nor necessary for most patients. A more realistic strategy is nested paired-compartment sampling in clinically appropriate subsets, combined with dense serial blood profiling in the broader cohort.

Sampling requirements differ by lineage and developmental question. For a neutrophil state, the marrow assay needs to capture the relevant granulopoietic progenitor and maturity compartments rather than rely on a generic HSPC signature. For monocyte maturation, progenitor-to-monocyte programs need to be measured alongside peripheral classical, intermediate and non-classical states and direct function. A generic marrow sample without lineage-resolved analysis will not resolve the attribution question.

### 10.2 Serial multi-omics and the limits of computational trajectory

Serial multi-omics can refine developmental hypotheses but cannot substitute for biological validation. scRNA-seq, scATAC-seq and CITE-seq can connect transcriptional programs, chromatin accessibility and surface phenotype; paired HSPC and mature-cell datasets can make developmental hypotheses more specific. Pseudotime and RNA velocity, however, remain model-based inferences. The post-septic MDSC study is instructive because it generates plausible transition structures while also revealing substantial heterogeneity (46). Trajectory inference becomes more persuasive when supported by compartment, time, surface phenotype and direct function, but it remains supportive rather than equivalent to lineage evidence.

### 10.3 Functional validation

The appropriate functional assay depends on the biological claim. A putative suppressor population requires direct assessment of suppressive capacity when feasible. A proposed antimicrobial defect is better examined with phagocytosis, killing, chemotaxis, oxidative burst or antigen-presentation assays than inferred from transcriptomic annotation alone. The discordance between CD10-defined maturity and T-cell suppression illustrates the value of this approach (16); LDN studies similarly show that direct functional assays can identify acquired suppression within a developmentally heterogeneous compartment (33, 34).

Functional assays themselves require context. Ex vivo suppression establishes biological capacity, but not necessarily the magnitude of the *in-vivo* effect. Conversely, a marker associated with outcome does not prove a specific cellular function. Mechanistically informative studies should align phenotype, molecular program, direct function and clinical trajectory instead of using one measurement as a surrogate for all others.

### 10.4 Clinical trajectory linkage

Clinical endpoints are most informative when they reflect the proposed mechanism. Mortality remains important but is biologically nonspecific. For low-response myeloid mechanisms, secondary bacterial or fungal infection, viral reactivation, duration of organ support, failure to recover immune competence, chronic critical illness/PICS and post-sepsis readmission may provide more direct biological linkage. Large mHLA-DR cohorts already illustrate the value of relating serial immune-state measurements to ICU-acquired infection rather than death alone (38). Future origin studies need to define the outcome a proposed state is expected to influence and account for competing processes capable of producing the same endpoint.

Repeated measurements also help guard against reverse interpretation. A cell state associated with mortality may be a marker of illness intensity or prolonged exposure rather than a mediator of death. Mechanism-linked outcomes and serial sampling improve temporal ordering and biological plausibility even when causal inference remains incomplete. In a literature rich in associations, that is more informative than requiring every newly defined state to predict mortality.

### 10.5 Standardization and replication

Standardization is essential. The same label can conceal different gates, sample-processing methods and analytical thresholds. mHLA-DR work shows that clinically useful standardization is possible while pre-analytic conditions and assay-specific thresholds still matter (59). For neutrophils and MDSC-like states, isolation method and suppression-assay configuration can materially alter phenotype or apparent function (48, 49). MDSC studies therefore require explicit phenotype definitions, direct functional criteria where the MDSC label is used, and restraint in applying that label to immature or low-density cells without functional support (22).

Replication is most informative when it tests biological equivalence rather than merely reproducing labels. A cluster name is comparable across datasets only when the underlying marker program, maturity context and function are also comparable. Multicenter harmonization of these layers would add more mechanistic information than additional cross-sectional state catalogues. A major remaining limitation is the lack of direct linkage among cell state, developmental origin and function.

## 11 Conclusions

Sepsis-associated myeloid dysfunction is not a single maturation defect and is unlikely to have one mechanistic origin. Human studies in clinical sepsis support altered hematopoietic and granulopoietic programs, while controlled human systemic-inflammation experiments provide cross-compartment evidence that monocyte maturation can also be perturbed. Independent human data show that circulating neutrophils and monocytes acquire epigenetic, metabolic, activation and suppressive states after marrow egress. Current evidence makes either a purely peripheral or a purely developmental model difficult to sustain, while remaining insufficient to quantify the contribution of each within the same patient and lineage.

Interpretation is clearest when measured variables are kept separate from inferred biology. Hematopoietic origin, developmental maturity, molecular state, function and patient endotype are different evidentiary layers. This resolves several recurring apparent contradictions: immature neutrophils are not synonymous with suppressive neutrophils; low-density neutrophils are not a developmental lineage; HLA-DR-low monocytes are not automatically immature or functionally validated M-MDSCs; and computational trajectories do not establish ancestry. The marrow-to-blood continuum is not a new taxonomy; it is a way to specify where evidence is direct and where attribution remains inferential.

Further progress requires longitudinal, cross-compartment studies with direct functional validation and mechanism-linked clinical outcomes. Until such data are available, the most defensible synthesis is that developmental contribution or remodeling and peripheral reprogramming both shape dysfunctional myeloid states in sepsis, while their relative roles remain unresolved. These source-attribution hypotheses are experimentally testable.

## References

[1] Singer M Deutschman CS Seymour CW Shankar-Hari M Annane D Bauer M . The third international consensus definitions for sepsis and septic shock (Sepsis-3). Jama. (2016) 315:801–10. doi: 10.1001/jama.2016.0287 26903338 PMC4968574

[2] Giamarellos-Bourboulis EJ Aschenbrenner AC Bauer M Bock C Calandra T Gat-Viks I . The pathophysiology of sepsis and precision-medicine-based immunotherapy. Nat Immunol. (2024) 25:19–28. doi: 10.1038/s41590-023-01660-5 38168953

[3] Moore AR Zheng H Ganesan A Hasin-Brumshtein Y Maddali MV Levitt JE . A consensus immune dysregulation framework for sepsis and critical illnesses. Nat Med. (2025) 31:4084–96. doi: 10.1038/s41591-025-03956-5 41028543 PMC12705441

[4] Zhu S He J . Inflammation-driven immune reprogramming in sepsis: from cytokine storm to immunoparalysis. Front Immunol. (2026) 17:1887033. doi: 10.3389/fimmu.2026.1887033 42495640 PMC13391346

[5] Reyes M Filbin MR Bhattacharyya RP Billman K Eisenhaure T Hung DT . An immune-cell signature of bacterial sepsis. Nat Med. (2020) 26:333–40. doi: 10.1038/s41591-020-0752-4 32066974 PMC7235950

[6] Kwok AJ Allcock A Ferreira RC Cano-Gamez E Smee M Burnham KL . Neutrophils and emergency granulopoiesis drive immune suppression and an extreme response endotype during sepsis. Nat Immunol. (2023) 24:767–79. doi: 10.1038/s41590-023-01490-5 37095375

[7] Yao R-Q Zhao P-Y Li Z-X Liu Y-Y Zheng L-Y Duan Y . Single-cell transcriptome profiling of sepsis identifies HLA-DRlowS100Ahigh monocytes with immunosuppressive function. Mil Med Res. (2023) 10:27. doi: 10.1186/s40779-023-00462-y 37337301 PMC10278311

[8] Supino D Davoudian S Silva-Gomes R Piovani D Garuti R Desai A . Monocyte-macrophage membrane expression of IL-1R2 is a severity biomarker in sepsis. Cell Death Dis. (2025) 16:269. doi: 10.1038/s41419-025-07597-x 40204720 PMC11982311

[9] Wu X Liu S Hu W Peng Q Huang W Ding J . From defense to dysfunction: Decoding the paradox of emergency granulopoiesis in sepsis pathogenesis. Research. (2025) 8:1011. doi: 10.34133/research.1011 41446870 PMC12722637

[10] Yang Y Zhang Y Wu J Liu Y Lei X . Decoding immune low-response states in sepsis: single-cell and 3D spatial transcriptomic insights into immunoparalysis. Front Immunol. (2025) 16:1696914. doi: 10.3389/fimmu.2025.1696914 41268561 PMC12626914

[11] Gao X Cai S Li X Wu G . Sepsis-induced immunosuppression: mechanisms, biomarkers and immunotherapy. Front Immunol. (2025) 16:1577105. doi: 10.3389/fimmu.2025.1577105 40364841 PMC12069044

[12] Wang X Huang Q Zuo Z Wang Z Zhang L Qian Z . Myeloid-derived suppressor cells in sepsis: drivers of persistent immunosuppression and targets for precision immunotherapy. Inflammation Res. (2026) 75:85. doi: 10.1007/s00011-026-02222-9 41917194

[13] Qian Z . Peripheral blood mononuclear cells in sepsis: immune trajectories, monocyte dysfunction, and translational biomarkers. J Inflammation Res. (2026) 19:1–23. doi: 10.2147/jir.s611267 42382469 PMC13317773

[14] Zhang R Lou X Huang H Zhou J Dai Q Zheng Z . Immune regulation and therapeutic targets in sepsis: insights from single-cell transcriptomics. Front Immunol. (2026) 17:1839417. doi: 10.3389/fimmu.2026.1839417 42292424 PMC13253376

[15] Keramati F Leijte GP Bruse N Grondman I Habibi E Ruiz-Moreno C . Systemic inflammation impairs myelopoiesis and interferon type I responses in humans. Nat Immunol. (2025) 26:737–47. doi: 10.1038/s41590-025-02136-4 40251340 PMC12043512

[16] Liu M Wang G Wang L Wang Y Bian Y Shi H . Immunoregulatory functions of mature CD10+ and immature CD10– neutrophils in sepsis patients. Front Med. (2023) 9:1100756. doi: 10.3389/fmed.2022.1100756 36687441 PMC9846122

[17] Marini O Costa S Bevilacqua D Calzetti F Tamassia N Spina C . Mature CD10(+) and immature CD10(-) neutrophils present in G-CSF-treated donors display opposite effects on T cells. Blood. (2017) 129:1343–56. doi: 10.1182/blood-2016-04-713206 28053192

[18] Kaneko T Stearns-Kurosawa DJ Taylor F Jr. Twigg M Osaki K Kinasewitz GT . Reduced neutrophil CD10 expression in nonhuman primates and humans after *in vivo* challenge with E. coli or lipopolysaccharide. Shock. (2003) 20:130–7. doi: 10.1097/01.shk.0000068326.68761.34 12865656

[19] Coudereau R Haem Rahimi M Lukaszewicz A-C Cour M Bidar F Argaud L . Immature neutrophils and myeloid-derived suppressor cells in sepsis: differences in occurrence kinetics. Crit Care. (2024) 28:7. doi: 10.1186/s13054-023-04781-3 38169413 PMC10759592

[20] Mehmeti-Ajradini M Bergenfelz C Larsson AM Carlsson R Riesbeck K Ahl J . Human G-MDSCs are neutrophils at distinct maturation stages promoting tumor growth in breast cancer. Life Sci Alliance. (2020) 3:e202000893. doi: 10.26508/lsa.202000893 32958605 PMC7536824

[21] Singel KL Emmons TR Khan ANH Mayor PC Shen S Wong JT . Mature neutrophils suppress T cell immunity in ovarian cancer microenvironment. JCI Insight. (2019) 4:e122311. doi: 10.1172/jci.insight.122311 30730851 PMC6483507

[22] Akkari L Amit I Bronte V Fridlender ZG Gabrilovich DI Ginhoux F . Defining myeloid-derived suppressor cells. Nat Rev Immunol. (2024) 24:850–7. doi: 10.1038/s41577-024-01062-0 38969773

[23] Mekata K Kyo M Tan M Shime N Hirohashi N . Molecular endotypes in sepsis: integration of multicohort transcriptomics based on RNA sequencing. J Intensive Care. (2025) 13:30. doi: 10.1186/s40560-025-00802-1 40448231 PMC12123803

[24] Peters-Sengers H Butler JM Uhel F Schultz MJ Bonten MJ Cremer OL . Source-specific host response and outcomes in critically ill patients with sepsis: a prospective cohort study. Intensive Care Med. (2021) 48:92–102. doi: 10.1007/s00134-021-06574-0 34902047 PMC8667541

[25] Park S Perumalsamy H Gerelkhuu Z Sunderraj S Lee Y Yoon TH . Phenotypic landscape of immune cells in sepsis: insights from high-dimensional mass cytometry. ACS Infect Dis. (2024) 10:2390–402. doi: 10.1021/acsinfecdis.4c00066 38850242

[26] Giamarellos-Bourboulis EJ Kotsaki A Kotsamidi I Efthymiou A Koutsoukou V Ehler J . Precision immunotherapy to improve sepsis outcomes. Jama. (2026) 335:775–86. doi: 10.1001/jama.2025.24175 41359996 PMC12687202

[27] Swann JW Olson OC Passegué E . Made to order: emergency myelopoiesis and demand-adapted innate immune cell production. Nat Rev Immunol. (2024) 24:596–613. doi: 10.1038/s41577-024-00998-7 38467802 PMC12942464

[28] Reyes M Filbin MR Bhattacharyya RP Sonny A Mehta A Billman K . Plasma from patients with bacterial sepsis or severe COVID-19 induces suppressive myeloid cell production from hematopoietic progenitors *in vitro*. Sci Transl Med. (2021) 13:eabe9599. doi: 10.1126/scitranslmed.abe9599 34103408 PMC8432955

[29] Lahoz-Beneytez J Elemans M Zhang Y Ahmed R Salam A Block M . Human neutrophil kinetics: modeling of stable isotope labeling data supports short blood neutrophil half-lives. Blood. (2016) 127:3431–8. doi: 10.1182/blood-2016-03-700336 27136946 PMC4929930

[30] Bongers SH Jukema BN Jansen A van Lier D Waalders N Kox M . *In vivo* cytokine adsorption reveals distinct neutrophil mobilization pathways during experimental human endotoxemia. J Innate Immun. (2026) 18:385–98. doi: 10.1159/000553231 42348490 PMC13485298

[31] Chen J Zhang J Chen S Qin J Hu X Xi S . CD121b-positive neutrophils predict immunosuppression in septic shock. Front Immunol. (2025) 16:1565797. doi: 10.3389/fimmu.2025.1565797 40230851 PMC11994419

[32] Guenther T Coulibaly A Velásquez SY Schulte J Fuderer T Sturm T . Transcriptional pathways of terminal differentiation in high- and low-density blood granulocytes in sepsis. J Inflammation. (2024) 21:40. doi: 10.1186/s12950-024-00414-w 39434093 PMC11492786

[33] Charoensappakit A Sae-khow K Vutthikraivit N Maneesow P Sriprasart T Pachinburavan M . Immune suppressive activities of low-density neutrophils in sepsis and potential use as a novel biomarker of sepsis-induced immune suppression. Sci Rep. (2025) 15:9458. doi: 10.1038/s41598-025-92417-7 40108283 PMC11923122

[34] Teague HL Knabe L S. Da Cruz R Yao X Allen KC Williams T . Low-density neutrophil heterogeneity and spleen tyrosine kinase as therapeutic targets in sepsis. JCI Insight. (2026) 11:e201057. doi: 10.1172/jci.insight.201057 42012886 PMC13313530

[35] Lundquist H Andersson H Chew MS Das J Turkina MV Welin A . The Olfactomedin-4-defined human neutrophil subsets differ in proteomic profile in healthy individuals and patients with septic shock. J Innate Immun. (2023) 15:351–64. doi: 10.1159/000527649 36450268 PMC10701106

[36] Leijte GP Rimmelé T Kox M Bruse N Monard C Gossez M . Monocytic HLA-DR expression kinetics in septic shock patients with different pathogens, sites of infection and adverse outcomes. Crit Care. (2020) 24:110. doi: 10.1186/s13054-020-2830-x 32192532 PMC7082984

[37] Bodinier M Peronnet E Brengel-Pesce K Conti F Rimmelé T Textoris J . Monocyte trajectories endotypes are associated with worsening in septic patients. Front Immunol. (2021) 12:795052. doi: 10.3389/fimmu.2021.795052 34912347 PMC8667763

[38] Monneret G Lafon T Gossez M Evrard B Bodinier M Rimmelé T . Monocyte HLA-DR expression in septic shock patients: insights from a 20-year real-world cohort of 1023 cases. Intensive Care Med. (2025) 51:1820–32. doi: 10.1007/s00134-025-08110-w 40986015 PMC12504386

[39] Miatello J Lukaszewicz A-C Carter MJ Faivre V Hua S Martinet KZ . CIITA promoter polymorphism impairs monocytes HLA-DR expression in patients with septic shock. iScience. (2022) 25:105291. doi: 10.1016/j.isci.2022.105291 36304101 PMC9593818

[40] Mathias B Delmas AL Ozrazgat-Baslanti T Vanzant EL Szpila BE Mohr AM . Human myeloid-derived suppressor cells are associated with chronic immune suppression after severe sepsis/septic shock. Ann Surg. (2017) 265:827–34. doi: 10.1097/sla.0000000000001783 27163951 PMC5102824

[41] Hollen MK Stortz JA Darden D Dirain ML Nacionales DC Hawkins RB . Myeloid-derived suppressor cell function and epigenetic expression evolves over time after surgical sepsis. Crit Care. (2019) 23:355. doi: 10.1186/s13054-019-2628-x 31722736 PMC6854728

[42] Waeckel L Venet F Gossez M Monard C Rimmelé T Monneret G . Delayed persistence of elevated monocytic MDSC associates with deleterious outcomes in septic shock: a retrospective cohort study. Crit Care. (2020) 24:132. doi: 10.1186/s13054-020-02857-y 32264937 PMC7137224

[43] De Zuani M Hortová‐Kohoutková M Andrejčinová I Tomášková V Šrámek V Helán M . Human myeloid‐derived suppressor cell expansion during sepsis is revealed by unsupervised clustering of flow cytometric data. Eur J Immunol. (2021) 51:1785–93. doi: 10.1002/eji.202049141 33788255 PMC8360154

[44] Jiang F Tu M Zhao J Wan Y Liu Z Zhang M . The clinical significance of myeloid-derived suppressor cells in patients with sepsis: a prospective cohort study. Sci Rep. (2026) 16:18988. doi: 10.1038/s41598-026-49604-x 42014467 PMC13275916

[45] Darden DB Bacher R Brusko MA Knight P Hawkins RB Cox MC . Single-cell RNA-seq of human myeloid-derived suppressor cells in late sepsis reveals multiple subsets with unique transcriptional responses: a pilot study. Shock. (2021) 55:587–95. doi: 10.1097/shk.0000000000001671 33021571 PMC8019679

[46] Barrios EL Leary JR Darden DB Rincon JC Willis M Polcz VE . The post-septic peripheral myeloid compartment reveals unexpected diversity in myeloid-derived suppressor cells. Front Immunol. (2024) 15:1355405. doi: 10.3389/fimmu.2024.1355405 38720891 PMC11076668

[47] Schrijver IT Karakike E Théroude C Baumgartner P Harari A Giamarellos-Bourboulis EJ . High levels of monocytic myeloid-derived suppressor cells are associated with favorable outcome in patients with pneumonia and sepsis with multi-organ failure. Intensive Care Med Exp. (2022) 10:5. doi: 10.1186/s40635-022-00431-0 35146585 PMC8831012

[48] Kremer V Godon O Bruhns P Jonsson F de Chaisemartin L . Isolation methods determine human neutrophil responses after stimulation. Front Immunol. (2023) 14:1301183. 10.3389/fimmu.2023.1301183 PMC10704165 38077317

[49] Negorev D Beier UH Zhang T Quatromoni JG Bhojnagarwala P Albelda SM . Human neutrophils can mimic myeloid-derived suppressor cells (PMN-MDSC) and suppress microbead or lectin-induced T cell proliferation through artefactual mechanisms. Sci Rep. (2018) 8:3135. doi: 10.1038/s41598-018-21450-6 29453429 PMC5816646

[50] Lorente-Sorolla C Garcia-Gomez A Català-Moll F Toledano V Ciudad L Avendaño-Ortiz J . Inflammatory cytokines and organ dysfunction associate with the aberrant DNA methylome of monocytes in sepsis. Genome Med. (2019) 11:66. doi: 10.1186/s13073-019-0674-2 31665078 PMC6820973

[51] Morante-Palacios O Lorente-Sorolla C Ciudad L Calafell-Segura J Garcia-Gomez A Català-Moll F . Jak2-stat epigenetically regulates tolerized genes in monocytes in the first encounter with gram-negative bacterial endotoxins in sepsis. Front Immunol. (2021) 12:734652. doi: 10.3389/fimmu.2021.734652 34867954 PMC8635809

[52] Mi Y Burnham KL Charles PD Heilig R Vendrell I Whalley J . High-throughput mass spectrometry maps the sepsis plasma proteome and differences in patient response. Sci Transl Med. (2024) 16:eadh0185. doi: 10.1126/scitranslmed.adh0185 38838133

[53] Shrestha D Pant BD Roychowdhury S Gandhirajan A Cross E Chhabria M . Immunometabolic chaos in septic shock. J Leukocyte Biol. (2025) 117:qiae211. doi: 10.1093/jleuko/qiae211 39340428 PMC11879763

[54] Shen R Jiang Y Liu G Gao S Sun H Wu X . Single-cell landscape of bronchoalveolar lavage fluid identifies specific neutrophils during septic immunosuppression. Adv Sci (Weinh). (2025) 12:e2406218. doi: 10.1002/advs.202406218 39887584 PMC11923989

[55] Samuelsen A Lehman E Burrows P Bonavia AS . Time-dependent variation in immunoparalysis biomarkers among patients with sepsis and critical illness. Front Immunol. (2024) 15:1498974. doi: 10.3389/fimmu.2024.1498974 39712015 PMC11659229

[56] Chenoweth JG Colantuoni C Striegel DA Genzor P Brandsma J Blair PW . Gene expression signatures in blood from a West African sepsis cohort define host response phenotypes. Nat Commun. (2024) 15:4606. doi: 10.1038/s41467-024-48821-0 38816375 PMC11139862

[57] Sun X-F Luo W-C Huang S-Q Zheng Y-J Xiao L Zhang Z-W . Immune-cell signatures of persistent inflammation, immunosuppression, and catabolism syndrome after sepsis. Med. (2025) 6:100569. doi: 10.1016/j.medj.2024.12.003 39824181

[58] De Zuani M Lázničková P Hortová Kohoutková M Bosáková V Andrejčinová I Vadovičová N . Sepsis induces long-term reprogramming of human HSPCs and drives myeloid dysregulation in sepsis survivors. J Inflammation. (2026) 23:4. doi: 10.1101/2024.12.14.628447 41593458 PMC12903497

[59] Quadrini KJ Patti‐Diaz L Maghsoudlou J Cuomo J Hedrick MN McCloskey TW . A flow cytometric assay for HLA‐DR expression on monocytes validated as a biomarker for enrollment in sepsis clinical trials. Cytometry Part B Clin Cytometry. (2021) 100:103–14. doi: 10.1002/cyto.b.21987 33432735

[60] Haem Rahimi M Conti F Llitjos JF Fleurie A Cerro V Venet F . Monocyte HLA‐DR expression as an enrollment biomarker in sepsis clinical trials: Evaluation of two sampling tubes and definition of respective clinical thresholds. Cytometry Part B Clin Cytometry. (2023) 104:468–70. doi: 10.1002/cyto.b.22133 37226415

[61] Leventogiannis K Kyriazopoulou E Antonakos N Kotsaki A Tsangaris I Markopoulou D . Toward personalized immunotherapy in sepsis: The PROVIDE randomized clinical trial. Cell Rep Med. (2022) 3:100817. doi: 10.1016/j.xcrm.2022.100817 36384100 PMC9729870

